# How do Tanzanian hospital nurses perceive their professional role? A qualitative study

**DOI:** 10.1002/nop2.139

**Published:** 2018-04-14

**Authors:** Ingrid Tjoflåt, Theodotha John Melissa, Estomih Mduma, Britt S. Hansen, Bjørg Karlsen, Eldar Søreide

**Affiliations:** ^1^ Faculty of Social Science Department of Health Studies University of Stavanger Stavanger Norway; ^2^ Haydom School of Nursing Haydom Tanzania; ^3^ Haydom Lutheran Hospital Haydom Tanzania; ^4^ Department of Research Stavanger University Hospital Stavanger Norway; ^5^ Stavanger University Hospital University of Stavanger Stavanger Norway

**Keywords:** hospital nursing care, nurses, professional role, qualitative research, Tanzania

## Abstract

**Aim:**

To describe the experiences of Tanzanian nurses, how they perceive their role as a professional nurse and their experience with nursing care in a general hospital.

**Design:**

This study is explorative, descriptive and qualitative.

**Methods:**

The data were collected in 2015 by means of 10 semi‐structured interviews and was analysed using qualitative content analysis.

**Results:**

The data analysis revealed two themes with corresponding sub‐themes related to Tanzanian nurses' perception with their professional role and experiences with nursing care: (1) Feeling professional pride; (2) Experiencing limitations and inadequacy. The findings indicate that the Tanzanian nurses possess a strong professional pride and commitment to serve and care for their patients. The nurses do their best to provide high ‐quality nursing care but are faced with staffing shortages and limited materials that are beyond their control. Such limitations leave them feeling unable to fulfil their role and responsibilities.

## 1 INTRODUCTION

Although the nurse workforce is an essential part of the Tanzanian health system, the health system suffers from a shortage of nurses and midwifes, as well as a deficit of basic medical equipment (Dolvo, 2007; Kwesigabo et al., 2012; The midterm analytical review of performance of the health sector strategic plan III 2009–2015, 2013). According to the World Health Organization, the nurse/midwife to population ratio is 0.4 per 1,000 in Tanzania and the figures vary between urban and the rural areas (WHO 2015). In comparison, Western countries have a far greater density of nurses and midwifes. For instance, Norway has a ratio of 17.3 nurses/midwifes per 1,000 and Germany has 11.4 per 1,000 (WHO 2015). In Tanzania, there are legal guidelines for nurses and midwifes covering many aspects of nursing care, education, research and management practice (Moyo, 2011; Tanzania Nursing and Midwifery Council, 2014). Nursing schools in Tanzania offer diploma programs, bachelor's programs and master's programs in nursing. The nursing programs reflect requirements of the Tanzanian Nursing and Midwifery Council and academic regulatory authorities for academic awards set by the government. The educational program is both theoretical and practical (Moyo, 2011; Tanzania Nursing and Midwifery Council, 2014). It is based on a scientific patient‐centred approach, nursing theory and nursing procedures, and traditional care of the sick based on culture, religious and spiritual beliefs. Respect, humanity and care are values reflected in the code of professional conduct for nurses and midwifes in Tanzania who are bound to these principles and expectations (Tanzania Nurses and Midwives Council, 2007). The Tanzanian code of conduct is adapted from the ICN Code of Ethics for Nurses (2000). However, to practice nursing care according to professional code of conduct can be challenging in a low resource setting. Previous studies from Tanzania and Uganda have demonstrated that the nurses try to do their best to provide adequate care to the patients, despite of problems with shortage of staff, overcrowded wards and lack of medical equipment (Fournier, Kipp, Mill, & Wallusimbi, 2007; Haggstrøm, Mbusa, & Wadensten, 2008; Harrowing & Mill, 2010). These studies have also revealed that in addition to the struggle of providing adequate care according to the nurses' professional and ethical standards, the nurses are accused for deficiencies that are beyond their control and this may lead to moral distress (Fournier et al., 2007; Haggstrøm et al., 2008; Harrowing & Mill, 2010). Moreover, nursing care in Tanzania has been reported to be medicalized and task‐oriented rather than patient‐centred (Juntunen & Nikkonen, 1996). On the other hand, research has revealed that patients of a HIV prevention program in rural Tanzania report good caring interactions and high quality of nursing care (Våga, Moland, Evjen‐Olsen, Leshabari, & Blystad, 2013). To the best of our knowledge, only three studies have examined the Tanzanian nurses' perceptions of their role and their experience with nursing care in different care settings. More research about these topics from the perspectives of the nurses is therefore needed. Thus, this paper describes the experiences of 10 Tanzanian nurses, how they perceive their role as a professional nurse and their experience with nursing care in a rural hospital. This study is a part of a larger study exploring the experiences of Tanzanian nurses who work in a rural hospital with foreign nurses from Western countries.

## 2 THE STUDY

### 2.1 Aim

The aim of this study is to describe how Tanzanian nurses perceive their professional role and their experiences with nursing care in a general hospital.

### 2.2 Design

A descriptive, explorative design that included semi‐structured individual interviews was applied. The data were collected over 2 weeks in 2015.

### 2.3 Setting

The study was carried out in a first level referral hospital in one rural setting in the northern parts of the United Republic of Tanzania. The hospital offers a wide range of services; curative, preventive and research services and implements a task‐orientated nursing care approach.

### 2.4 Participants

A total of 11 local nurses working in the hospital were invited to take part in the study and 10 of these agreed. The recruitment of participants followed a strategy of purposeful sampling by selecting nurses with experiences anticipated to benefit the study (Polit & Beck, 2012). Moreover, proficiency in English was considered as a selection criteria (Gray, 2014). The local identified co‐investigator, who was familiar with the study and the inclusion criteria, selected the study participants. The sample comprised seven female and three male nurses with a median age of 45 years (34–53 years). The sample included nurses who worked in the maternity, paediatric, surgical and medical wards, and the intensive care unit. Their average working experience was 18 years, with a range 4–31 years. Three participants had a bachelor's degree and seven participants had a Diploma in Nursing. Five nurses held senior supervision positions in the hospital ward, while five nurses were working as regular ward nurses.

### 2.5 Ethical considerations

We obtained permission to conduct the study from the National Institute for Medical Research (NIMR) (23 February 2015: NIMR/HQ/R.8a/Vol. IX/1915), the Tanzania Commission for Science and Technology (COSTECH, NO.2015‐122‐NA ‐2015‐78) and the reference person in the selected hospital in the United Republic of Tanzania. In addition, the Norwegian Social Science Data Services (no: 38988) approved the study. Information letters in both English and Kiswahili that outlined the purpose, scope, content, confidentiality and practicalities of the study were provided to the participants before the interviews started. Participants were informed that their participation was voluntary and that they could withdraw from the study at any time. All participants signed letters of consent.

### 2.6 Data collection

The first author collected the data through semi‐structured interviews using open‐ended questions and following a topical interview guide (Polit & Beck, 2012). The guide focused on nursing care in the hospital ward with questions that inquired about the reasons why the nurses chose their profession, what they thought was important to the role of a nurse and their thoughts about nursing care in the hospital ward. The guide was also translated to Kiswahili, which is the local language. The local co‐investigator who spoke the local language was present at all the interviews. Each interview started with an introduction, a presentation of the participants and an oral explanation of the confidentiality, practicalities and purpose of the study. It was emphasized that we wanted to hear their experiences and that there were no right and wrong answers to the questions. The interviews were conducted in English. Some of the participants needed to clarify some of the questions in Kiswahili with the local co‐investigator during the interviews. When this happened, the first author asked to have the sentences translated to English. To ensure that information provided to the nurses was well‐understood, the first author repeated some of their answers. The interviews were conducted in a separate hospital room with no disturbance or interruption. They were audiotaped and transcribed verbatim.

### 2.7 Data analysis

The interview text was analysed by qualitative content analysis that was performed in several steps (Graneheim & Lundman, 2004). After reading the first part of the transcript interviews several times to obtain an understanding of the whole, the meaning units that emerged from the text were identified and provided a condensed process that preserve the core meaning. Moreover, the condensed meaning units were organized into subthemes and themes. Common patterns were compared to identify the similarities and differences in the nurses' experiences in their professional nursing role and with the nursing care in the hospital. The lead author first analysed the data independently, after which the fourth co‐author read the transcribed interviews. Consequently, the first and fourth co‐author discussed the various steps in the analysis and the emerging themes. The analysis identified two main themes related to the experiences of nursing role and care.

## 3 FINDINGS

The analysis resulted in the identification of two main themes with corresponding sub‐themes related to the nurses' descriptions of their professional nursing role and the nursing care: (1) Feeling professional pride; and (2) Experiencing limitations and inadequacy. Table 1 presents an overview of the main themes together with their corresponding sub‐themes. The two themes, each of which was based on subthemes are presented in more details below. Quotations from the interviews are added to give the meaning of the text.

**Table 1:** Overview of main themes with corresponding sub‐themes in the qualitative content analysis

Main themes	Sub‐themes
Feeling professional pride	Motivated to help
	Pleased to provide patient‐centred care
Experiencing limitations and inadequacy	Limited human and material resources
	Feeling of inadequacy

### 3.1 Feeling professional pride

This theme emerged from the nurses' descriptions of how they perceived their professional role when caring for patients in everyday nursing practice in the hospital ward and is illustrated by the following two subthemes: 1) being motivated to help; and 2) being pleased to provide patient‐centred care.

The participants stated that their motivation for becoming a nurse was to help and care for sick patients. One nurse expressed the job's appeal: “I like to work with the patients, to help the sick people, to talk to them spiritually, mentally and physically, I like to care for sick people” (nurse 3).

Another motivational aspect for becoming a nurse was the opportunity to provide care of high quality to the patients. Two nurses told that they had wanted to become a nurse since their childhood, as illustrated by one nurse who recounted their initial motivation: “Even when I was young I told my father that I have to become a nurse. I decided to be a nurse to save the women” (nurse 1).

Many of the nurses emphasized essential aspects of nursing care to help patients who cannot self‐manage due to illness. One nurse discussed the nature of nursing care: “Nursing care is to be patient‐centred, to take care of the patient, to love and to be close to the patient” (nurse 7).

Another participant illustrated that being a nurse was an innate feeling. She wanted to serve people who cannot self‐manage due to their illness.

Moreover, several nurses emphasized the importance of providing optimal care to help patients recover from illness, as expressed in the following quotation: “The important thing is to provide the best care for the patients according to their physical, psychological and sometimes spiritual and social needs so they can recover” (nurse 4).

Two of the nurses mentioned that they perceived the nursing care in the hospital to be good. One expressed the nature of their satisfaction with the nursing care: “I am happy with the nursing care, happy because I meet my goals. I talk with the clients and I provide the care” (nurse 3). Other nurses described that they wanted to provide good care and worked hard to achieve this, as illustrated by one of them: “Good care is to work hard to meet the clients' needs, to make sure that information needed is provided and that the deliveries are successful” (nurse 2).

Many of the participants described the varied responsibilities of working as a nurse in the wards, such as performing certain skills like checking vital signs, documenting nursing care, giving medication, preparing the patients for surgery, giving injections, dressing wounds and following the doctors' orders. Some of these characteristics are illustrated in the following quotation from a nurse: “My nursing activities are to give the patients medication, to prepare them for surgery and to receive them from surgery and postoperative care” (nurse 9).

Other nurses described nursing care as being more focused on guiding patients and relatives and the importance of a good approach towards the patients. One participant said: “The women coming for delivery are not equal. As a nurse you have to be polite and guide the women” (nurse 1).

### 3.2 Experiencing limitations and inadequacy

This theme reflects on the limitations and the sense of inadequacy that nurses experienced when performing nursing care in the ward. This is described by the following two subthemes: 1) limited human and material resources; and 2) feeling of inadequacy.

All the nurses described that the limitations and sense of inadequacy they encountered in their everyday nursing practice were mainly related to lack of staff, too many patients in the wards and a lack of equipment. One nurse discussed the impact of the shortages: “Sometimes we are few nurses in the ward and we do not reach all of our goals. When we are few nurses, the work becomes difficult and sometimes we do not have enough equipment” (nurse 9). Another nurse revealed that such shortages would require nurses to skip certain duties: “We omit…documentation and other activities due to too many patients and very few nurses” (nurse 10).

Due to lack of staff and shortage of equipment, the nurses told that it was a challenge to provide adequate care and they described the feeling of inadequacy that arose from not being able to fulfil their responsibilities. This is illustrated in the next quotations:

“When taking care of many patients at the same time, the care was not as good as I expected” (nurse 5).

The medicine is not available…and you cannot help the patient. The patients are suffering. These are the challenges, big challenges. You are looking at the patient, who has difficulties in breathing, but you cannot help. It is a big challenge' (nurse 6).

However, even with the shortage of staff they tried to provide the best possible nursing care.

## 4 DISCUSSION

The present findings reveal how 10 Tanzanian nurses perceived their professional nursing role and their experiences with nursing care in a hospital. Two main themes emerged from the interviews. The first indicates that by being motivated to help the patients and pleased with providing patient‐centred care, the nurses felt professional pride. The second theme indicates that limited human and material resources and feeling inadequate led to experience of limitations and inadequacy.

The findings indicate that the nurses were proud of their role of providing nursing care to the patients. The nurses stated that it was essential for them to give patient‐centred care, to meet the patients' needs and to give good care. The nurses' strong commitment to care for patients was an important motivational aspect in joining the profession. Some of them had even had this motivation from childhood. These findings are in line with the goals of the education of Tanzanian nurses and are reflective of the values of the professional code of conduct (Moyo, 2011; Tanzania Nurses and Midwives Council, 2007; Tanzania Nursing and Midwifery Council, 2014). Moreover, the findings are in accordance with previous research that indicated that nursing was a passion and that nurses are proud of their work (Haggstrøm et al., 2008; Harrowing & Mill, 2010).

Although most of the nurses described nursing care to be a patient‐centred approach that included physical, psychological, social and spiritual care, the findings also show that nursing care was described as performing certain skills and following doctors' orders. This may reflect that nurses also had a task‐orientated nursing care approach when working in the wards, which is consistent with a previous study from Tanzania, (Juntunen & Nikkonen, 1996). Additionally, the nurses participating in the study were all working in the same hospital where a task‐orientated nursing care approach was implemented. This could explain the fact that it was difficult to perform a patient‐centred nursing care approach with few nurses and overcrowded wards.

In this study, many of the nurses experienced several challenges when performing nursing care that are linked to factors such as a shortage of staff, overcrowded wards and lack of medical equipment. Nevertheless, these factors were often beyond the nurses' control, as indicated in previous studies (Fournier et al., 2007; Harrowing & Mill, 2010). Nurses in the current study stated that they were unable to provide optimal quality nursing care due to the many challenges they encountered, although they tried to do their best to help the patients. This may indicate that quality nursing care is difficult to perform when working in hospitals without enough workplace resources and necessary medical equipment. These finding are in line with previous research that showed that quality nursing care for patients can only be provided by nurses who have adequate workplace resources concerning both necessary medical equipment and nurses (Fournier et al., 2007; Haggstrøm et al., 2008; Harrowing & Mill, 2010). Studies have revealed that staff shortages and lack of medical equipment are the most direct effect of migration of nurses from Sub‐Saharan countries to Western countries. This migration might affect the already overburdened health systems to deliver quality nursing care (Dolvo, 2007; Willis‐Shattuck et al., 2008). The paradox of the situation is that the nurses from Sub‐Saharan countries with low density of nurses are providing nurses to Western countries with a far greater density of nurses and midwifes (Dolvo, 2007). Moreover, previous studies among East Africa nurses have shown that nurses who experience such shortages are at special risk of suffering moral distress and burnout (Fournier et al., 2007; Haggstrøm et al., 2008; Harrowing & Mill, 2010; van der Doef, Mbazzi, & Verhoeven, 2011).

### 4.1 Study limitations

It is essential that the voices in this study reflect the experiences of a small group of Tanzanian nurses with good proficiency in one rural hospital. The findings will therefore not be representative of Tanzanian nurses in general. Moreover, the first author did not speak the local language and some of the participants had now and then difficulties with expressing themselves in English. To minimize this weakness, the local investigator clarified sentences in the local language during the interviews and the first author repeated the participants' answers to verify that it was well‐understood. Another factor that might have influenced the data's trustworthiness could be the fact that the first author had a Western background and that the local investigator was a nurse teacher. This may have caused the participants to answer in a way they thought were expected, as they wanted to express their experiences in a positive light (Polit & Beck, 2012). Finally, it is possible that the credibility of the study may have been affected as we did not give the participants the opportunity to check our interpretation of the data. However, it should be noted that the findings section mainly contains manifest findings presented in the themes, which may have enhanced transparency.

## 5 CONCLUSION

The present study describes the experiences of Tanzanian nurses in their professional nursing role and the nursing care in a rural hospital. The findings demonstrate that the nurses are proud of being a nurse and have a strong commitment to serve and care for the patients. The nurses were doing their best to perform good nursing care, but found this difficult due to shortages of nurses and material limitations beyond their control. The findings also reveal that the nurses described a sense of inadequacy due to being unable to fulfil their role and responsibilities due to the limitations.

### 5.1 Implications for practice and future research

The findings in this study may provide important knowledge guiding nursing care in a resource‐limited country like Tanzania as well as informing the nursing profession globally about how the Tanzanian nurses experience their role and care. An adequate amount of resources and equipment seems to be paramount in providing quality care. Therefore, comprehensive and integrated strategies involving individual, institutional, community and government levels should be called for to meet the challenges of such workplace deficiencies. Further research is needed before conclusions that are more definite can be drawn about how nursing care is experienced in resource‐limited countries from both the perspectives of nurses and patients.

## DISCLOSURE

The authors have confirmed that all authors meet the Nursing Open for authorship credit.

## CONFLICT OF INTEREST

No conflict of interest has been declared by the authors.

## AUTHOR CONTRIBUTIONS

IT, Study conception/design; IT. TM, Data collection; IT, BSH, Data analysis; IT, Manuscript writing; EM, BSH, TM, BK, ES, Critical revisions for important intellectual content.
